# A Review on the Impact of Bedside Echocardiography in Managing Critically Ill Children in the Pediatric Intensive Care Unit

**DOI:** 10.7759/cureus.69769

**Published:** 2024-09-20

**Authors:** Sri Sita Naga Sai Priya K, Amar Taksande

**Affiliations:** 1 Pediatrics, Jawaharlal Nehru Medical College, Datta Meghe Insititute of Higher Education and Research, Wardha, IND

**Keywords:** bedside echocardiography, doppler ultrasound, hemodynamics, pediatric critical care, ultrasound waves

## Abstract

Over the past three decades, a variety of non-invasive hemodynamic devices have been developed. However, none of the existing methods, such as transthoracic echocardiography, esophageal Doppler ultrasound, plethysmography, thoracic impedancemetry, or sublingual capnography, fully embody the ideal characteristics of reliability, reproducibility, rapid response, ease of use, comprehensive safety, affordability, and continuous monitoring capacity. Among these, echocardiography stands out as a particularly effective approach, meeting many of these criteria due to its widespread availability, relative ease of use, and critical role in detecting anatomical abnormalities and basic changes in myocardial function. It is frequently used in pediatric intensive care units to assess the structure and function of the heart muscle. The effectiveness of echocardiography in pediatric critical care is also constrained by the need for high-quality imaging and accurate interpretation. Currently, there is a notable lack of literature on the application of echocardiography in pediatric critical care. This study seeks to evaluate the existing scientific evidence regarding the effectiveness of echocardiography as a tool for monitoring hemodynamics in pediatric critical care settings.

## Introduction and background

Effective management of critically ill pediatric patients in intensive care units necessitates accurate diagnosis and prudent timing of intervention. Pediatric intensivists now regularly include echocardiography as a standard part of their patient assessments in pediatric intensive care units (PICUs) [1]. Echocardiography is an effective method that enables direct viewing of the heart, guiding patients' hemodynamic condition at the bedside. This hemodynamic estimate provides physicians with information to guide treatment methods, such as volume resuscitation, initiation, termination, or change of vasopressor therapy. It also helps determine whether a prompt referral to a specialist is essential for cardiac or surgical intervention. While there is substantial evidence in the cardiology literature supporting the use of echocardiography, data specific to its application in critical care is less established but continues to grow [2].

The indications for bedside echocardiogram in training in intensive care and neonatal echocardiography (TINEC) encompass the evaluation of heart function, pulmonary hypertension, significant pericardial effusion, and tamponade. Therefore, it functions as a reference for therapies in severely critical pediatric patients [3]. Echocardiography enables real-time assessment of hemodynamic status, including cardiac function and preload. This technique helps estimate cardiac output and fluid responsiveness, measure pulmonary artery systolic pressure, and perform serial evaluations [4]. Hence, the present study was undertaken to assess the various studies to determine the precision of point-of-care limited echocardiography performed at the patient's bedside and to verify the therapeutic interventions carried out following the echocardiographic assessment. Furthermore, analysis was carried out to identify the reasons for conducting echocardiographic evaluations in critically ill pediatric patients, examine the connection between the need for these evaluations and the primary illness, and assess the clinical severity.

The evaluation of the patients' hemodynamics is a routine task in the pediatric critical care unit. The clinical examination both guides and determines its completion. The desire to provide patients with the least intrusive coverage feasible is a persistent concern. Haemodynamic monitoring, especially if it is invasive, must carefully consider the benefits and risks of initiating this procedure in a vulnerable patient. Over the past 30 years, a multitude of non-invasive haemodynamic instruments have been created. An ideal solution should possess the qualities of reliability, reproducibility, fast response time, ease of use, complete safety, affordability, and continuous monitoring capability. None of the available tools, such as esophageal Doppler ultrasound, transthoracic echocardiography, non-invasive cardiac output (NICO), thoracic impedancemetry, plethysmography, or sublingual capnography, possess all the ideal features. However, transthoracic echocardiography is the tool that most effectively supports monitoring hemodynamic parameters [5].

## Review

Principle of echocardiography

Echocardiography utilizes ultrasound technology to generate images of the heart and vascular systems. Ultrasound waves, with frequencies above 20,000 cycles per second-beyond the range of human hearing-are produced by a machine comprising a central processor and a transducer. The transducer, containing piezoelectric crystals, converts mechanical energy (sound) into electrical energy and vice versa. These crystals generate mechanical energy through sinusoid and l cycles of compression and rarefaction, directing a focused beam towards the heart. This beam travels in a straight line until it encounters structures with different acoustic impedances, such as the boundary between blood and tissue, where a portion of the energy is reflected back to the transducer while the remainder continues forward. These reflections, known as ultrasound echoes, are essential for constructing the cardiac image. The proportion of transmitted and reflected acoustic energy depends on tissue characteristics. However, obstacles like air, bone, bandages, or foreign objects that interfere with acoustic signal reflection can significantly reduce image clarity, a common challenge in intensive care settings [6].

Echocardiography offers essential hemodynamic insights, including measurements of cardiac output (CO), end-diastolic volume, and assessments of both global and regional ventricular function, along with the detection of valvular abnormalities [7-11]. The systolic function (SF) can be evaluated by calculating the shortening fraction and ejection fraction using M-mode or two-dimensional imaging. Doppler-derived aortic velocity curves are also employed to assess systolic function by analyzing parameters such as peak velocity, acceleration time, ejection time, isovolumic contraction time, and the velocity-time integral. These assessments produce indices like the peak rate of acceleration and mean acceleration, which are closely related to systolic function. Ratios such as acceleration time to ejection time and isovolumic contraction time to acceleration time serve as markers of ventricular function [7,9]. Diastolic function is assessed using established parameters such as the E wave, A wave, E/A ratio, E', A', and the myocardial performance index (MPI) or Tei index, all measured via Doppler echocardiography [9,11]. These measurements specifically examine ventricular relaxation, or the ATP-dependent phase of ventricular diastole.

Indications

Echocardiography is expected to become a standard practice in PICUs in the future. Bedside echocardiogram is a valuable diagnostic tool in the PICU that may rapidly and noninvasively assess the great veins, ventricular size, and contractility. It is portable and allows for early diagnosis of reversible and time-dependent disorders [12,13]. Echocardiography is employed in the pediatric intensive care unit to diagnose pulmonary hypertension, patent ductus arteriosus, evaluate hemodynamics, assess cardiac function, and identify pericardial effusion and cardiac tamponade [4].

In a study by AI-Ghwass et al. [14] the primary purpose for echocardiography was left ventricular function assessment in 68 cases (34%). In 108 patients (54%) who had echocardiographic examination, no difference in management was detected, while 92 patients (46%) received additional information or decision aid. In a study carried out by Miller et al. [15] comparing fluoroscopy with 2D echocardiography in 36 juvenile cardiothoracic patients to evaluate aberrant hemidiaphragm motion, the authors discovered that echocardiography had a sensitivity of 100% and a specificity of 81%, whereas fluoroscopy had a sensitivity of 100% and a specificity of 74%. Their study findings thus confirmed the efficacy of echocardiography in evaluating diaphragm function. Furthermore, the authors proposed that when the diaphragm is clearly visible through echocardiography, the inclusion of a fluoroscopic study would not significantly enhance its clinical utility.

In a study by Abd El Massih et al. [16], bedside echocardiography was conducted on 271 critically ill patients in the neonatal and pediatric intensive care units at Cairo University Children's Hospital. Each patient underwent a detailed clinical examination, chest X-ray, and echocardiogram, with results compared across these modalities. Pulmonary hypertension was the most frequently observed condition in the neonatal intensive care unit (35.8%), while impaired cardiac contractility was most common in the pediatric intensive care unit (33.1%). In the neonatal unit, the primary indication for bedside echocardiography was screening for hemodynamically significant patent ductus arteriosus in preterm infants (n=73, 42%), whereas in the pediatric unit, the assessment of cardiac function was most prevalent (n=60, 61.9%). Additionally, during cannulation, ultrasound helps verify correct vascular access and the proper placement of the inflow cannula. While on extracorporeal membrane oxygenation (ECMO), echocardiography is essential for assessing ventricular loading conditions, monitoring hemodynamics, checking cannula placement, and detecting potential thrombi in the heart or aorta to prevent complications. Echocardiography is vital throughout all stages of ECMO [17].

Pediatric echocardiography is indicated for a wide range of symptoms and signs, including cyanosis, failure to thrive, exercise-induced chest pain or syncope, respiratory distress, heart murmurs, congestive heart failure, irregular arterial pulses, or cardiomegaly. These symptoms may suggest various types of structural congenital heart disease, such as atrial and ventricular septal defects, ventricular outflow tract obstruction, mitral and tricuspid regurgitation, Transposition of great arteries, double outlet right ventricle and complex anomalies like dextrocardia. Echocardiography is also recommended in clinical situations involving certain syndromes, a family history of inherited heart disease, or extracardiac anomalies associated with congenital heart disease, even in the absence of specific cardiac symptoms or signs. 

In addition to the indications mentioned above, echocardiography in the PICU has been employed for tasks such as verifying the position of a cannula during pediatric ECMO, as well as evaluating aberrant motion of the hemidiaphragm that is clinically significant [18].

Bedside echocardiography: assessing clinical conditions and guiding treatment

Şahin et al. [19] studied echocardiographic indications and treatment approaches in critically ill children, analyzing the relationship between the Pediatric Risk of Mortality Score III (PRISM) and the need for echocardiography. In a prospective study of 140 children, including 75 mechanically ventilated and 65 spontaneously breathing, echocardiography led to changes in treatment strategies for 64.6% of patients. The study found a significant correlation between higher PRISM scores and the necessity for echocardiographic evaluation, particularly in mechanically ventilated children (p<0.001). However, in a recent study by Al-Ghwass et al. [14] there was no discernible relationship between the PRISM score and the efficacy of echocardiographic evaluation. Furthermore, a significant association was seen between the effective value of echocardiography and the therapy of patients with primary cardiac illness (p-value = 0.001), shock (p-value = 0.001), and hypoxia (p-value = 0.009).

Pershad et al. [20] evaluated the effectiveness of emergency physician-performed bedside limited echocardiography (BLEEP) for estimating left ventricular function (LVF) and inferior vena cava (IVC) volume as indirect preload measures. They found a strong correlation between BLEEP and pediatric echocardiography provider (PEP) estimates, with a mean discrepancy of 4.4% in SF estimation and a strong agreement in IVC volume estimation. Rabah et al. [21] reported that in 81.8% of cases analyzed over 24 months in a PICU, novel echocardiographic findings significantly influenced clinical decisions, resulting in changes to medication and procedures.

Orme et al. [22] performed a total of 258 echocardiograms on 217 patients, with critical care consultants conducting 86.8% of these. Among the echocardiograms, 72.4% were transthoracic (TTE) and 27.8% were transesophageal (TOE). The diagnostic information provided influenced management in 51.2% of cases, including changes in fluid administration, inotropic therapy, and treatment limitations. Additionally, Vignon et al. [12] found that hand-held echocardiograms (HHE) have the potential to accurately detect various conditions in mechanically ventilated critically ill patients, showing promise as an alternative to traditional TTE.

Kutty et al. [23] investigated how echocardiography findings led to changes in clinical management, analyzing and categorizing these modifications. They conducted 416 echocardiograms on 132 patients, with 31% performed on infants under 30 days old and a median patient age of 103 days. Their study found that 63% of echocardiograms confirmed initial diagnoses, 24% altered them, and 13% revealed new, unrelated findings; notably, management adjustments were often associated with urgent echocardiograms and unexpected discoveries, though no significant correlation was found with patients under 30 days old.

Bhadane et al. [24] found that out of 100 children admitted to the PICU, 83% were classified as critically ill. They observed left ventricular hypertrophy (LVH) and a high left ventricular mass index (LVMI) in 22.9% of children with abnormal renal profiles, hypertension, and hypocalcemia. Additionally, significant findings included 88.7% with left ventricular diastolic dysfunction, 68.7% with abnormal right ventricular function, and fluid-responsive states indicated by high IVC-CI and IVC-DI in a majority of cases, with 69.9% requiring medical intervention based on 2D echocardiography; 12% of the children died during the study.

Şahin et al. [19] reported that 64.6% of patients received vital or additional information from echocardiography. Stanko et al. [25] found that in 27% of cases, referring physicians deemed clinical information insufficient for diagnosis and treatment, prompting requests for TTE in 73% of studies, which led to diagnostic changes in 29% and management changes in 41% of cases. Ali et al. [26] similarly found that over 50% of echocardiograms revealed new findings that influenced treatment decisions, underscoring the value of routine echocardiographic assessments in intensive care settings.

In a study by Ranjit et al. [27] over 28 months, 22 of 37 patients with septic shock remained in shock despite receiving 60 ml/kg of fluid and dopamine/dobutamine infusions. Among these patients, 12 were in warm shock and 10 in cold shock, with six of the cold shock patients showing an atypical pattern of wide pulse pressures on invasive monitoring. Echocardiography identified underlying pathophysiological issues in these patients, revealing uncorrected hypovolemia in 12 and reduced left or right ventricular function in 10, leading to necessary adjustments in their treatment.

Kobr et al. [28] highlighted the benefits of using frequent bedside echocardiography for comprehensive hemodynamic monitoring in critically ill children, noting that it enhances heart function evaluation compared to traditional methods. Research by Gargani et al. [29] and Murphy et al. [30] supports this approach, showing that tracking changes in cardiac performance indices offers valuable insights when conducted by a skilled intensivist. Echocardiography can estimate pulmonary artery systolic pressure (PASP) using the pressure gradient between the right ventricle and right atrium, measured via tricuspid regurgitation, and is particularly useful in diagnosing pulmonary hypertension, commonly seen in pediatric intensive care settings [31-33].

Echocardiographic assessment is a crucial tool, especially in mechanically ventilated children, with its importance growing as the severity of their condition increases. Basic training in echocardiography for intensivists is crucial and must be enhanced and supported in the care of critically ill patients. Similarly, Khilnani et al. [34] emphasized that intensivists should receive formal training in bedside echocardiography, considering the following challenges: the need for a learning curve to master the technique, variability in observations by different practitioners, and interpretation difficulties in the presence of confounding factors such as patients who are spontaneously breathing versus mechanically ventilated, elevated intraabdominal pressure, low tidal volumes, and reduced lung compliance.

Several ultrasound courses, either offered by universities or private sector organizations, are currently developing with a specific focus on pediatric patients. One example is an annual basic ultrasound course organized by the Society of Critical Care Medicine and the Indian Society of Critical Care Medicine. The crucial matter that remains unattended is the presence of numerous obstacles hindering the utilization of bedside echocardiography in children who are critically ill. In addition , some cardiologist hold differing opinions regarding the use of limited-focused emergency echocardiography [34].

Transthoracic echocardiography is a competence that can be attained to a specific level by the majority of intensivists. Nevertheless, it is crucial to keep in mind that intensivists should refrain from doing echocardiograms autonomously unless they have had thorough training and official accreditation. There is no existence of a "fundamental echo" in children. A novice operator may produce subpar images and misinterpret high-quality ones. The errors can have significant clinical implications. Hence, it is imperative to possess adequate training and skill in order to effectively perform this valuable surgery at the patient's bedside [35].

Echocardiography, both in diagnosis and in surveys, surpasses all other non-invasive techniques for assessing hemodynamics in terms of the quality and quantity of information obtained. it is also highly useful for identifying septic thrombus or infective endocarditis. It plays a crucial role in assessing pericardial effusion in patients with rheumatological diseases. Additionally, it is valuable in diagnosing conditions such as rheumatic heart disease, multisystem inflammatory syndrome in children (MIS-C), dengue, and acute pancreatitis. The acquisition of knowledge regarding fundamental functions such as contractility evaluation, cardiac output, and cardiac and vascular filling, which are essential for initiating a treatment, is rather proficient [34]. In the end, monitoring is only valuable when the physician detects a particular value or pattern and has the necessary competence to respond properly [36].

## Conclusions

In summary, bedside echocardiography offers essential information that is not apparent during a clinical examination and serves as a straightforward, non-invasive tool for identifying the underlying causes in critically ill pediatric patients. Furthermore, bedside echocardiography often leads to a change in diagnosis and management. These echo findings allow for adjustments in therapy that would not be possible based solely on clinical examination. Echocardiography provides insight into the underlying disturbed pathophysiology and justifies the need for treatment adjustments. It is important for bedside clinicians to have proficiency in fundamental echocardiographic assessment, as it has the potential to significantly impact the treatment course for patients, even though the specific beneficial parameters are currently unknown. Pediatric cardiologists can provide training and supervision for emergency physicians conducting bedside echocardiography for evaluating hemodynamic parameters and detecting other abnormalities in emergency scenarios.
